# Prototyping a Web-of-Energy Architecture for Smart Integration of Sensor Networks in Smart Grids Domain

**DOI:** 10.3390/s18020400

**Published:** 2018-01-30

**Authors:** Víctor Caballero, David Vernet, Agustín Zaballos, Guiomar Corral

**Affiliations:** Engineering Department, Universitat Ramon Llull (URL), La Salle, 08022 Barcelona, Spain; dave@salleurl.edu (D.V.); zaballos@salleurl.edu (A.Z.); guiomar@salleurl.edu (G.C.)

**Keywords:** smart grids, sensor networks, web of things

## Abstract

Sensor networks and the Internet of Things have driven the evolution of traditional electric power distribution networks towards a new paradigm referred to as Smart Grid. However, the different elements that compose the Information and Communication Technologies (ICTs) layer of a Smart Grid are usually conceived as isolated systems that typically result in rigid hardware architectures which are hard to interoperate, manage, and to adapt to new situations. If the Smart Grid paradigm has to be presented as a solution to the demand for distributed and intelligent energy management system, it is necessary to deploy innovative IT infrastructures to support these smart functions. One of the main issues of Smart Grids is the heterogeneity of communication protocols used by the smart sensor devices that integrate them. The use of the concept of the Web of Things is proposed in this work to tackle this problem. More specifically, the implementation of a Smart Grid’s Web of Things, coined as the Web of Energy is introduced. The purpose of this paper is to propose the usage of Web of Energy by means of the Actor Model paradigm to address the latent deployment and management limitations of Smart Grids. Smart Grid designers can use the Actor Model as a design model for an infrastructure that supports the intelligent functions demanded and is capable of grouping and converting the heterogeneity of traditional infrastructures into the homogeneity feature of the Web of Things. Conducted experimentations endorse the feasibility of this solution and encourage practitioners to point their efforts in this direction.

## 1. Introduction

Contrary to the rapid evolution experienced in the last decade of Information and Communication Technologies (ICTs), electric power distribution systems have remained exceptionally steady for a long time. Therefore, the Smart Grid concept, the addition of a telecommunication infrastructure to the electrical domain, has enabled a plethora of new services and opportunities (e.g., accurate grid monitoring, real-time energy consumption, and customer-side generation, etc.), which are currently driving a major revolution in the energy sector [1,2]. In fact, Smart Grids are conceived to improve traditional electric power networks in several dimensions [3] such as information, business models and scalability, in order to meet the highest standards of power quality and thus ensuring that the electric power grid is cost effective and sustainable [4].

Moreover, new agents and components have been raised inside this new paradigm. For example, the prosumer (load producer and load consumer) as the last link in the electricity value chain is easily the most important creator of value within the smart grid. Prosumers will be given a more active role in new business model generation. The most important connections for prosumers in the electricity value chain are the distributed system operator or the aggregator/retailer in addition to the Energy Services Company/provider (ESCO) or Virtual Power Plants (VPPs), which are also new components in the energy market value chain [3]. Specifically:

- The aggregator controls low voltage power that is transferred to the usual places, where it is consumed with metering and billing functionalities.
- The Energy Service Companies (ESCOs) will play an important role in the future electricity market as energy-oriented commercial businesses. ESCOs can be described as specialists in providing a broad range of comprehensive energy solutions, including the design and implementation of energy saving projects, energy conservation, energy infrastructure outsourcing, power generation and energy supply and risk management.
- A VPP can either operate large numbers of relatively small-sized generators, responsive loads and storage units on behalf of owners (in this case, prosumers) or operate its own Distributed Energy Resources (DERs).

Those new agents and components of the Smart Grid could become the most enriched elements due to their added information and technology features. However, the upgraded features of the end user side come at the cost of requiring more technology in order to make them usable. Due to the complexity (i.e., stringent levels of service reliability and availability), magnitude (i.e., large-scale areas), and new agent profiles inherent to Smart Grids, practitioners have recently addressed the digital transformation of power electric networks by proposing flexible and future internet based architectures [4].

As far as the communication and network protocols’ field is concerned, the Smart Grid has an inherent heterogeneous nature which forces system architects to consider different telecommunication technologies. Smart Grids are deployed in hostile wireless communication environments [5]; hence, channel status aware protocols [6] (also referred to as cognitive radio techniques) are needed to reduce communication delay [5], meet Quality of Service (QoS) needs in terms of delay, bandwidth, and data reliability, improve energy harvesting techniques [6], and provide reliable distributed sensing [7] in order to minimize interoperability issues between heterogeneous communication networks [8].

The Internet of Things (IoT) is introduced as the natural evolution of Internet, as it refers to the heterogeneous agglomeration of devices connected to the network. This evolution has succeeded thanks to the advances of both silicon, which have made it possible for increasingly smaller and smaller computing units to be embedded into everyday devices, as well as advances in low-power wireless protocols for such devices. The ICTs for the Smart Grids represent a good example of this heterogeneity, where different devices, both sensors and actuators, from different vendors and using different protocols, have to work together to achieve an objective: the integration of energy and smart services. Due to this heterogeneity issue, the Web of Things (WoT) [9] is presented as an abstraction layer that enables the homogeneous interaction between devices of different kinds using web technologies (previous work in JITEL 2017 [10]). In this sense, the grouping of the application of the methodologies provided by WoT to the management of Smart Grids under the term Web of Energy (WoE) [11,12] is proposed. In previous works [12], some proposals were introduced in this sense, however, it soon became apparent that another approach was needed for the development of an architecture for the WoE.

The paper is organized as follows: Section 2 describes the related work regarding Smart Grids, Web of Things, and Web of Energy, detailing their value proposals and associated challenges. Section 3 explains the problem of the direct integration of Smart Grids, Web of Things, and their communications protocols in order to compose the Web of Energy. Section 4 illustrates the hybrid architecture that is proposed that covers the needs of a Smart Grid infrastructure and provides the advantages of integrating the Smart Grids’ ICT with the Web of Things, that is, the Web of Energy. In Section 5, we conduct experiments to evaluate the performance of the prototype system and discuss the results. Section 6 concludes the article. Finally, Section 7 discusses further work.

## 2. Related Work

### 2.1. Smart Grids and Web of Energy

Over the last decade, Smart Grids have led the revolution of the electrical grid, transforming it into a set of automated and efficiently controlled processes by the incorporation of ICTs. Smart Grids promote electrical energy management in a distributed and flexible manner. However, the current management systems are (i) centralized regarding their management; (ii) located in independent locations between them; and (iii) managed by fragmented applications, without integration between them and only intercommunicated thanks to specific communication channels, which are generally proprietary.

The main goal of Smart Grids is to provide better services and features (also known as smart functions), for both consumers and for producers and prosumers. In addition, the increased use of distributed and renewable energy generation requires changes in the electricity management system. It is necessary to improve automation systems, distributed intelligence, real-time data mining and management to improve network control functions, simplify configuration and also reduce system recovery and self-healing times.

Recent advances in Smart Grids have explored the feasibility of considering the electrical power distribution networks as a particular case of the IoT. Certainly, this specific domain poses appealing challenges in terms of integration, since several distinct smart devices from different vendors (wired or wireless sensors, smart meters, distributed generators, and so on), often using proprietary protocols and running at different layers, must interact to effectively deliver energy and provide a set of enhanced services and features [8]. Although the latest developments of the IoT field have definitely contributed to the physical connection of such an overwhelming amount of smart devices, several issues have arisen when attempting to provide a common management and monitoring interface for the Smart Grid [13].

To solve the integration of heterogeneous devices, the use of the concept of the WoT [9] has been proposed to access multiple devices using the same interface provided by web technologies, implying uniformity in communication protocols (HTTP and WebSockets) and uniformity in the model of data representation and device discovery (Web Thing Model [14] and Semantic Web [15]). This concept is, so far, difficult to apply in real scenarios involving energy management due to the risk of creating new security vulnerabilities, the lack of devices that implement standards of the WoT and the opposition of the industry of energy to include external modules or devices in their proprietary systems.

The objective of the work presented in this paper is to create an architecture based on the IoT paradigm to manage the storage and communication needs of the Smart Grids and at the same time link the Smart Grids with the end user through the methodologies of the WoT. For this, a bidirectional Human-To-Machine interface is established, inspired by the WoT that allows the ubiquitous control of the energy systems, the WoE [11].

In this way, the WoE could represent an opportunity for the electric companies to have virtual representations of their more flexible devices (based on software, updatable, configurable, and with the possibility of deploying new applications on top of them), enabling a low-cost distribution and management of the electric network [16]. The WoE facilitates the sharing of data from different devices (charging points for electric vehicles, smart metering, or monitoring of substations) with third parties. In addition, the use of web technologies allows the creation of multiplatform visualization tools without installation cost, being able to offer simple and usable graphic interfaces for a greater adoption for the Distribution Systems Operator (DSO) or any user interested in the consumption or production of energy (prosumer). Thus, we can identify different fields related to energy management in which the WoE would be very useful:

- Remote access form substations to central servers.
- Management and monitoring of DERs.
- Supervisory Control and Data Acquisition (SCADA) distribution to secondary substations.
- Electrical Vehicle Supply Equipment (EVSE) management.

### 2.2. Web of Things

Although the initial predictions of one trillion devices connected to the Internet by 2015 [17] were quickly lowered to 26 billion by 2020 [18] and 20.8 billion in the same year [19], it is evident that the number of devices connected to the Internet is increasing every day. How to access all these sensors and actuators through a uniform interface is undoubtedly one of the biggest challenges of the IoT. Currently, the IoT is divided into self-contained areas, that is, there are proprietary solutions that help the integration of a specific set of devices, but this structure is far from the overall integration planned for the IoT. For this reason, the use of existing web technologies for the global integration of devices is proposed. The basic prerequisites for enabling the IoT devices on the web are two: (1) minimum capacity for data processing and (2) connectivity to the network (it is not necessary to connect directly to the Internet).

The model proposed by the WoT [9] aims to solve the challenge of the heterogeneity of the IoT devices. This is principally achieved by generating translators or mappings between the language spoken by each device (communication protocol and data format) and the language that we can consider “universal” due to its widespread use: web technologies. Specifically, we have the HTTP protocol and the RESTful APIs [20]. These translations enable the devices to speak the same language, which makes them accessible in a homogeneous way to either human or computerized actors, like other devices. The actions to be taken on these devices can be both to act and to sense.

If we focus on the data flow, it is composed of two states that can be understood as a cycle: from heterogeneity to homogeneity and from homogeneity to heterogeneity.

- From heterogeneity to homogeneity: a device sends data captured with a specific format and protocol through a WoT translator, which translates the data into a common format and the protocol into HTTP.
- From homogeneity to heterogeneity: the actor receives this data and decides to act on the device, for example, changing its configuration. It then sends an instruction using the HTTP protocol and a format common to the WoT translator which, in turn, translates the protocol and format of the data into the specific protocol and format of the device.

## 3. From the Smart Grid to the Web of Energy

### 3.1. Smart Grid Architecture Modules

**Figure 1:**
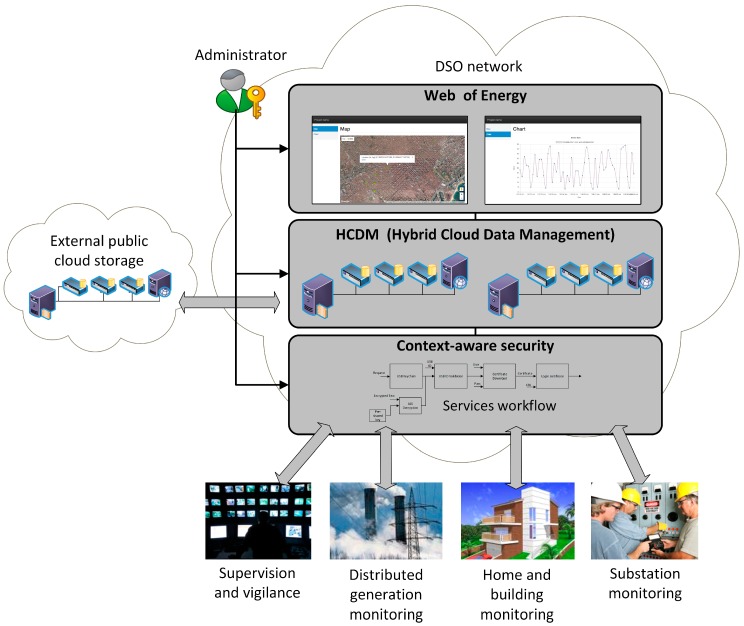
Main modules: Context-aware security, Hybrid Cloud Data Management and Web of Energy.

Our proposed Smart Grid architecture is composed of three main independent modules (see Figure 1). Each module is detailed in what follows.

- Context-aware security. This module aims to individually provide the needed security level for the proper operation of every smart function. For instance, for the use case of Smart Metering, this module can update all the encryption keys of the Smart Grid once an unauthorized access to the metering infrastructure has been detected.
- Hybrid Cloud Data Management: This module provides a data storage and processing system that intrinsically adapts to the Smart Grid’s topology in a scalable and flexible way. Also, it implements an algorithm (also referred to as orchestrator) to decide whether collected data should be stored at the private cloud or could be placed at the public cloud. Such decisions are taken considering the smart functions’ requirements (e.g., reliability, delay, and cybersecurity) associated with the collected data [12].
- Web of Energy: This module provides a ubiquitous (i.e., web based) monitoring interface [12] that enables a seamless management of the whole IoT architecture of the Smart Grid. In addition to providing a mechanism to communicate humans and machines, it also enables the interactions among those IoT resource-constrained and small devices (i.e., machine to machine) through the HTTP protocol. Note that the aforementioned context-aware security module might decide to add an extra layer of security by switching from HTTP to HTTPS in accordance with the device features, network status, and smart function under execution demands. This is done through an open API that both couples and decouples all the modules. Hence, this module also acts as a bridge between the distributed storage layer—that takes care of all the Smart Grid’s Big Data concerns—and the context-aware security layer—that gives the necessary access control and cybersecurity mechanisms.

### 3.2. Web of Things Architecture

**Figure 2:**
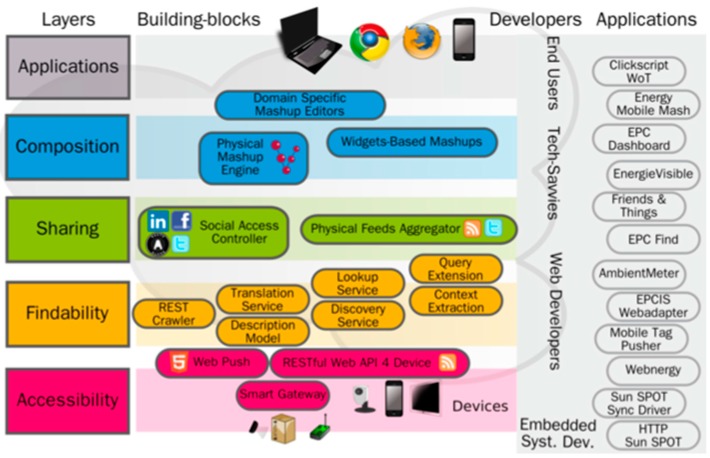
Web of Things layer model proposed in [9].

The concept of WoT is developed mainly in the works of Dominique Guinard [9] and Vlad Trifa [21], where the WoT and the methods of enabling devices from IoT to WoT, respectively, are presented. In [9] the WoT is organized into four layers, each with a specific function. However, these layers do not follow an isolation and encapsulation model between non-contiguous layers, as is the case with the OSI (Open Systems interconnection) or TCP/IP (Transmission Control Protocol/Internet Protocol) model but, on the contrary, the applications can be built on top of each one (Figure 2), since all of them are part of the application level of the two network models mentioned above. Next, the different layers of the WoT proposed in [9] are presented.

The functions of each of these layers are the following:

1. Accessibility: It enables a consistent access to all types of IoT devices by exposing their functionalities through a RESTful HTTP API.
2. Findability: It provides the discovery of the representations of the different devices, uniformly modeling the access method (through the HTTP protocol) and establishing relations between them at the moment of their representation.
3. Sharing: It is in charge of preserving the privacy between the device representations. It also manages the authentication and authorization to access the representations by actors who do not own the device.
4. Composition: It enables the integration between the different representations of the devices that, ultimately, allows the integration between the different functionalities of the physical devices.

#### 3.2.1. Accessibility Layer

The accessibility layer acts as an interface between the IoT and web technologies. Therefore, it is the closest layer to the heterogeneity of IoT protocols, such as MQTT (Message Queuing Telemetry Transport) [22] or CoAP (Constrained Application Protocol) [23], among others. In this layer, solutions are found in the form of a proxy or bridge between the IoT and HTTP protocols.

Two basic WoT-enabling methodologies are considered. Embedding web servers to devices, or the creation of gateways [24] that serve as aggregators or proxies between IoT protocols and web protocols. Cloud solutions are also found in this layer, generally transverse to more layers of the WoT, such as ThingWorx [25], Watson IoT Platform from IBM [26], Octoblu [27] or EVRYTHNG [28], among others. These cloud solutions offer IoT-WoT translation gateways.

#### 3.2.2. Findability Layer

This layer is composed of the technologies that allow for the exploration and searchers of the different devices exposed to the WoT. Those technologies allow the publication of structured and interconnected data. The concept of REST (Representational State Transfer) [20] and Linked-Data [29] is applied to interconnect different device representations (usually in the form of URLs—Uniform Resource Locators). Semantic Web technologies [15] such as RDF (Resource Description Framework) [30], RDFa (Resource Description Framework in Attributes) [31] or OWL (Web Ontology Language) [32] are used to provide semantic meaning to both representations and connections with other representations, allowing, for example, the exposition of the information to search engines.

#### 3.2.3. Sharing Layer

This layer groups all the methods that allow authentication and authorization of different actors to perform an action on the virtual representation of the device and, ultimately, on the physical device. It groups simple authentication and authorization methods from user authentication and password to the use of more advanced protocols such as API or OAuth keys.

A middleware called *Social Access Controller* is proposed in [33]. By combining the OAuth APIs of different social networks and the access by simple credentials to the devices (e.g., user and password), it allows to: (i) preserve the privacy of the physical devices; (ii) take advantage of the structure and function of social networks to authenticate the different actors potentially interested in participating in the actions that can be performed on the device representation (iii) integrate the representations of the devices in social networks, creating a Social Web of Things; and (iv) enable the publication of data aggregation using data syndication protocols like Atom [34].

#### 3.2.4. Composition Layer

This layer enables the composition of the different functionalities exposed by the device’s representations. Taking into account that each representation is accessible through the HTTP protocol, it is easy to program a script so that the different devices act in a coordinated way. The elaboration of this idea consists of providing the web user (human) with a visual interface to compose different device relationships. Solutions such as ThingWorx Composer [35], NODE-Red [36] from IBM or Octoblu [27] or IFTTT [37] exemplify the composition of physical devices (physical mashups).

### 3.3. Web of Things and Web of Energy Protocols

The main element of the WoT proposal is the use of web technologies for the transmission of information. As far as web protocols are concerned, there is the HTTP(S) protocol and the WebSocket (WS) or WebSocket Secure (WSS) protocol. HTTP is a request/response protocol while WebSocket allows a bidirectional connection between the client and the server.

However, many devices do not have the necessary features to directly access the web through HTTP or WebSocket. To be able to connect to the web, they need to use gateways that are responsible for translating the heterogeneity of the IoT protocols to the homogeneity of the WoT. To do this, it is necessary to establish translation bridges or mappings from IoT to WoT protocols and vice versa.

For example, both CoAP and MQTT are currently two of the most promising standard IoT protocols. CoAP, an open standard, was designed specifically for the IoT and to be directly compatible with HTTP [38]. It is a protocol based on request/response through UDP (User Datagram Protocol) packets and follows the same schemes as HTTP, allowing the creation of REST resources. The MQTT protocol, designed by IBM, is now also an open standard, but follows a publisher/subscriber paradigm over TCP, so it becomes more complicated to establish a translation bridge between HTTP-REST and MQTT [39].

Table 1 shows the differences and similarities between CoAP, HTTP, MQTT and WebSocket protocols. As mentioned, the mapping between CoAP and HTTP is direct, since the topology of both is request/response (Req/Resp). On the other hand, the topology between HTTP and MQTT is different, so the translation between these two protocols is more difficult to achieve. Another example of (almost) direct mapping is between WebSocket and MQTT, since the bidirectional communication of WebSocket can be considered a particularity of the publisher/subscriber protocol (Pub/Sub) of MQTT. Thus, HTTP and WebSocket are the two web technologies with different topologies that allow translation between IoT protocols with request/response and bidirectional topologies respectively. Table 1 also shows other protocols that can be part of the IoT, which have more similarities with HTTP or WebSocket according to their topology in the same way that occurs with CoAP and MQTT.

In addition to the two protocols already presented in Table 1 and many other proprietary and open-source protocols for IoT, protocols that are more adapted to the specific needs of electric utilities are beginning to emerge, particularly in sectors where the infrastructures are becoming obsolete. However, those protocols become more difficult to adapt to the WoE because: (1) its specificity reduces the interest of third parties of contributing to the exposure of the devices (or sets of them) to the WoT and, therefore, the companies should invest more capital in the mapping and (2) in case it is of interest for the company to expose some of their devices, the WoT adds a stack of new technologies, opening new security holes in their systems. These challenges are significant in the WoE, because the systems involved cover a large number of devices that use very specific protocols adapted to their needs.

### 3.4. Complementing the Web of Things Architecture

The main goal of this section is to list the key features that a WoT architecture should comply with. These features are the following:

- Although certain IoT devices can support HTTP stacks, there are many of them that can only support lighter protocols due to their limited resources. Although ideally a narrow range of IoT protocols (e.g., CoAP and MQTT) would facilitate the integration of the devices to the Internet and the Web, a basic and indispensable requirement for the devices to be part of the Web is that they can be connected to the Internet.
- The architecture must provide abstractions so that developers can interact independently with the devices of the communication protocol, either towards the heterogeneity of the IoT protocols or towards the homogeneity of the WoT protocols.
- The architecture must be able to scale horizontally and provide self-healing for its systems. It must permit the on-demand deployment of resources.
- In the WoE architecture, the devices have to become virtual objects [40] or things, although virtual objects created from aggregations of other virtual objects can also be created.
- Each virtual object will be accessible from an HTTP REST interface and, in case the features of the virtual object allow it, from a WebSocket link.
- The architecture must allow executing authentication and authorization protocols towards the different devices involved.

**Figure 3:**
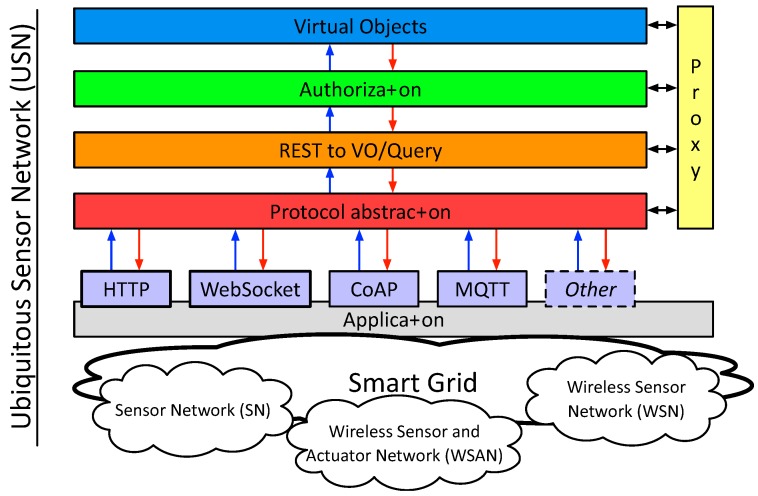
Proposed architecture for the Web of Energy.

In our proposal, we have identified five layers that compose the architecture of the WoE (Figure 3). Although they have a deep relationship with the layers proposed in [9] and other emerging architectures [41], some differences can also be identified, mainly due to the fact that the layers presented below are more focused on the development of the WoE architecture.

The description of the different layers of the proposed WoE architecture is the following:

1. Protocol Abstraction Layer: The objective of this layer is to provide an abstraction layer for developers to interact with physical devices, both for developers of architecture functionalities at the more internal layers as well as for application developers for Smart Grid that, as already specified, group different protocols. The goal is not only to expose the IoT devices as HTTP REST resources, but to provide developers with abstraction mechanisms of both HTTP and the IoT protocols. In this sense, the premise exposed in [9] of enabling access to physical devices through an HTTP interface is maintained.
2. REST to VO/Query: The purpose of this layer is to translate the different URIs generated from the information of physical devices to actions on virtual objects or Things. In this way, we do not act directly on physical devices but through virtual objects. This layer also includes an interface for more complex queries about a *Thing* or the relationships of different *Things*.
3. Authorization: This layer is responsible for requesting and granting access to virtual objects or *Things*.
4. Virtual Objects: When physical devices must perform actions on other physical devices they will send the instruction against a virtual representation (virtual object or *Thing*). The objective of this virtual representation is to increase the resources and functionalities of physical devices. We cannot provide a detailed list of added features, as they are specific to each solution. Even so, our goal is to provide a method (code injection) so that these functionalities can be added dynamically. Some common functionalities are related to reasoning (artificial intelligence) or caching.
5. Proxy Layer: The architecture of the WoT presented in [9] proposes that all representations of the devices use web technologies (e.g., HTTP) to communicate and expose their characteristics and achieve homogeneous access to the devices, both between devices and between devices and humans. However, using web technologies for all types of communication between specific servers of the WoT will not always be optimal. The motivation of this layer is therefore to show that efficient communication between servers can be carried out through a protocol that is not within web technologies. However, it is important to mention that even those services should have an HTTP interface for the integration in the WoT.

Finally, the application layer conceptualizes all those applications that are likely to interact with the architecture. Within the range of applications supported by the WoT and, therefore, the IoT, those applications of Smart Cities and Smart Grids are grouped. Specifically, the generation of a Ubiquitous Sensor Network [8] is an application that includes the agglomeration of wired and wireless networks of sensors and actuators.

### 3.5. Web of Energy Implementation

#### 3.5.1. First Approach: PHP Implementation

Our first goal in [12] was to create a high-performance WoT architecture, while experimenting with the most used web programming language up to that point, PHP (PHP: Hypertext Preprocessor), to see if it could be facilitated to the many web programmers in PHP [42,43] the programming interfaces for the WoT, thus achieving greater adoption in less time. However, the results were not positive for the following reasons:

- PHP web servers follow a very strict execution model that hinder the use of web technologies such as WebSocket. This is because WebSocket connections are permanent, whereas PHP servers delimit the connection with the client until a certain time limit or until the script execution is finished, since its main execution model is “load, execute, and die”. There are alternatives to this execution model like the one implemented in React PHP [44] (reactor pattern), but this library is not available for large-scale production environments such as WoT.
- PHP, an interpreted language, is slower than compiled languages. In addition, with the execution model discussed above, main developers have never been concerned with optimizing the generated instructions or eliminating multiple memory leaks. Now, with the arrival of PHP 7, an improvement in performance has been achieved [45], although the other reasons discussed in this list are still valid.
- There are very few libraries focused on functionalities that involve mathematical calculations intrinsic to Machine Learning or distributed computing models. This is due, once again, to the idiosyncrasies of this type of languages, which are mainly used to serve dynamic web pages.

#### 3.5.2. Second Approach: Actor Model Implementation

PHP is not efficient enough nor does it have the necessary tools for a large-scale implementation of an infrastructure for the WoT, as it was concluded in [12]. Once we discarded the use of PHP to develop the infrastructure of the WoT, we looked for a programming language that had the performance, the ecosystem and the necessary tools to develop such infrastructure. The JVM (Java Virtual Machine) provides a stable execution environment (since it is supported by Oracle) and many tools and libraries (protocols, web frameworks, and Machine Learning libraries) are available for this platform.

On the other hand, we were interested in finding a programming model that facilitated the creation of distributed, flexible, and self-healing systems, mandatory features for the WoE. For this reason, the Actor Model [46,47] seemed to be the ideal model. This model or paradigm of programming facilitates the creation of systems with those requirements and, in addition, thanks to the fact that it allows concurrency, it is capable of taking advantage of the different device computing cores [48].

The basic principles of operation of this model are that when an Actor receives a message, it can:

- Send messages to other Actors
- Create new Actors
- Designate how to manage the next received message

**Figure 4:**
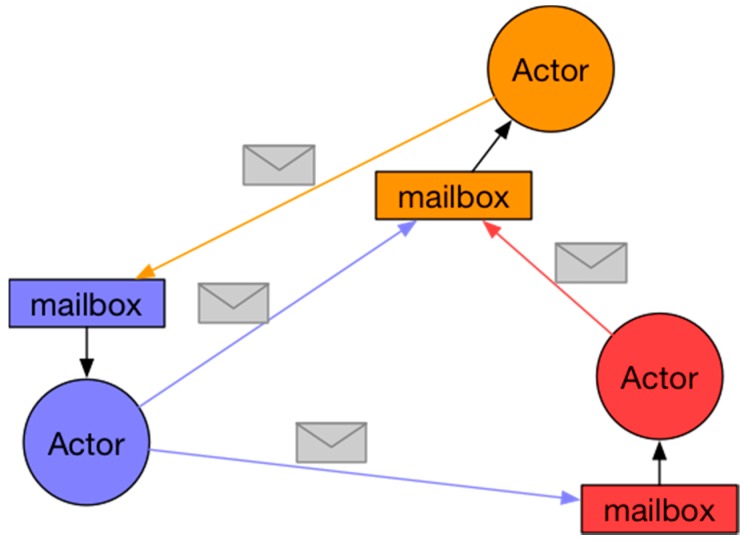
Representation of the messaging system in the Actor Model.

Several Actors can run concurrently although an Actor can only process one message at a time. That is why each Actor receives messages in a totally asynchronous way, storing them in a local message queue. Figure 4 represents this idea.

As can be observed, when an Actor receives a message, it is stored in a mailbox that is specific to each Actor and inaccessible to the rest of the Actors. This mechanism maintains a private status and isolates an Actor from the rest.

Although the Actor Model can be used to create agents and, therefore, Multi-Agent Systems (MASs), this article focuses on the exposure and use of the model per se in IoT or WoT.

In [49], authors benefit from this model to enable features such as multi-cloud and multi-tenure for IoT devices. They take advantage of the few resources that an actor needs to operate by compartmentalizing the physical devices in different software modules (actors) that are isolated and connected to different cloud infrastructures and have different owners.

In [50], the use of CAF (C++ Actor Framework) is proposed to provide developers with a high-level Operating System abstraction framework to develop IoT applications. The authors also emphasize the properties of abstraction, distribution and flexibility of this computation model.

Thanks to the principles on which the Actor Model is built, we can obtain interesting properties for the WoE infrastructure.

- Distribution and Flexibility: One of the basic principles of the model is the creation of new actors. This facilitates the use of computing resources not only in the same node but distributed in different nodes since the communication is asynchronous and transparent between local and remote node actors. Besides being able to create new actors, they can also be eliminated and, therefore, permit the management of the use of resources.
- Self-Healing: This property is not based on any principle of the theoretical Actor Model, but all the libraries that implement this model also implement it since the creation of Erlang [51], due to its great practical use. By definition, the state of an actor is isolated in that actor and can only communicate with the outside through messages. Self-healing takes advantage of this isolation principle to manage the failure of a given actor. For example, Akka [52] implements parental supervision. The creation of actors by another actor results in the creation of a hierarchy with a parent actor. In the event of the failure of a “child” actor, the “parent” actor can decide how to manage this failure: restart it or not, for example. Thanks to the isolation between actors and the supervision between actors, execution errors can be isolated and managed in a controlled manner. Actor state recovery mechanism involve event sourcing [53] techniques, where messages that change the state of the actor are logged and replayed when the actor restarts.

Thanks to the basic principles of an actor and the properties that derive from them, we also propose that each actor (or group of them) represents the virtualization of a physical device, increasing the resources and functionalities of such a device and isolating the execution failures of the other actors, that is, of the system. Actually, this proposal is not novel because there are different references [46,47] that propose this same use for the IoT. Our intention is to provide a working framework and an implementation for the WoE that meets its expectations. It is also our objective to promote the use of the Actor Model because it provides important abstractions to develop distributed systems, flexible, self-healing, and designed to achieve maximum and optimal use of resources thanks to parallel computing.

## 4. Prototypes, Initial Model, and Emulation Setting

In this section, we present the successfully implemented prototypes that are governed by the techniques analyzed above.

### 4.1. Layer Prototypes

Prototypes of some of the layers described above have been implemented for the proof of concept. Specifically, the Virtual Objects Space and the Protocol Abstraction layer have been implemented. Also, a publisher/subscriber protocol for the proxy layer has been considered to communicate two remote servers. Below both are detailed:

- Virtual Objects Space: Each physical device is represented by a virtual object and this in turn represented by one or more actors. This layer defines the logic and abstractions necessary for each device to be represented by one or more actors, in such a way that the data flow, taking as a starting point the reception of data from a protocol of what we consider to be heterogeneous or IoT (e.g., MQTT), is transformed into a homogeneous protocol (proxy layer) so that it can be understood by the other parts of the architecture.
- Proxy layer: According to the needs of this proof of concept, the proxy layer has been implemented through a publisher/subscriber protocol. In this way, the virtual objects that represent the devices can publish the captured data and can subscribe to messages also collected by other devices or to action messages sent by other devices.
- Protocol Abstraction Layer: Contains the implementations that interface between different protocols such as MQTT, WebSocket, or HTTP.

**Figure 5:**
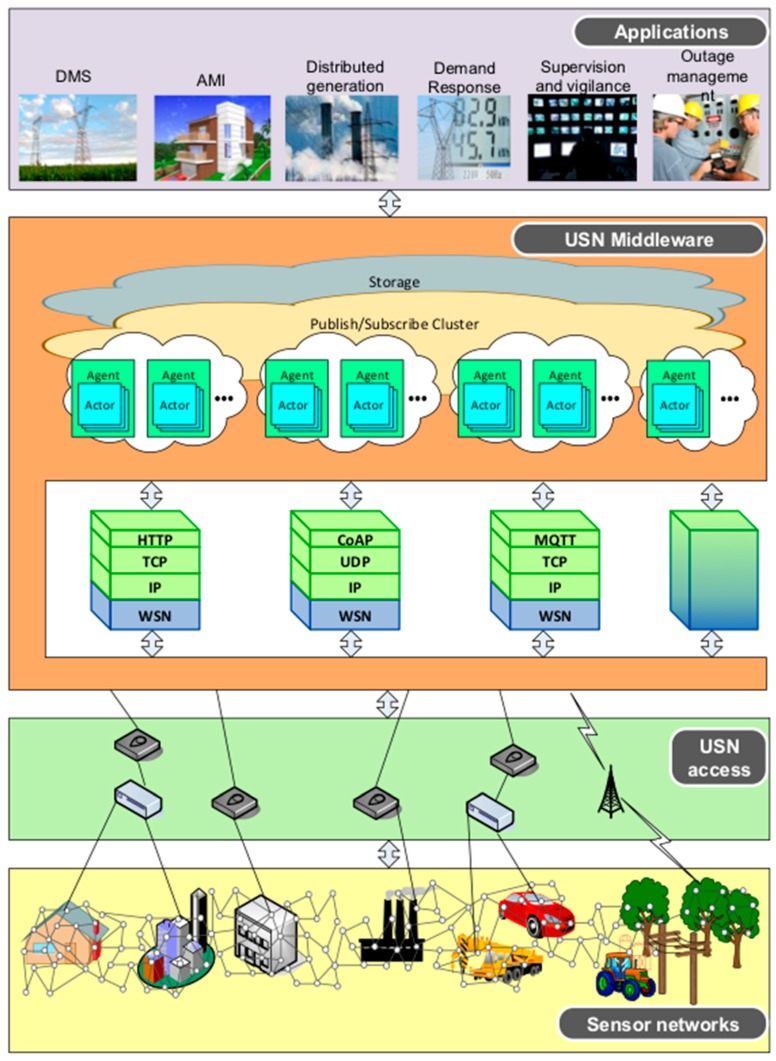
Layer schematic for a Ubiquitous Sensor Network (USN) applied to Smart Grids.

Figure 5 shows the interaction between the different layers in a Smart Grid. We find, considering a top-down vision, (i) the application layer with the intelligent functions of Smart Grids; (ii) the middleware proposed in this paper; (iii) the accessibility layer to the Ubiquitous Sensor Network (USN) with the aggregators of multiple sensors; and finally, (iv) the sensor networks. Intentionally, this schematic has a strong resemblance to Figure 2 shown in [8]. In this case, we have focused on the middleware development for a USN.

### 4.2. Initial Model

There are three well-differentiated sections in our implementation model. These sections are the following:

- MQTT Section: It consists of those devices that communicate through the MQTT protocol and the servers or services responsible for translating MQTT into an “understandable” protocol.
- Virtual Objects Space and Proxy Layer: This section is made up of those modules responsible for representing each physical device through a virtual object and the publisher/subscriber protocol.
- HTTP/WebSocket: Analogous to the MQTT section, it includes those devices that communicate with the architecture through web protocols and the services responsible for translating these protocols into a protocol “understandable” by the internal layers of the architecture.

**Figure 6:**
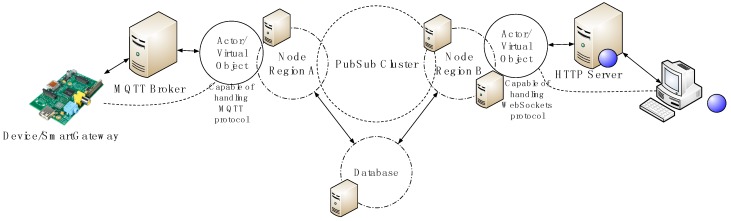
System design and architecture.

The architectural model is depicted in detail in Figure 6.

**Figure 7:**
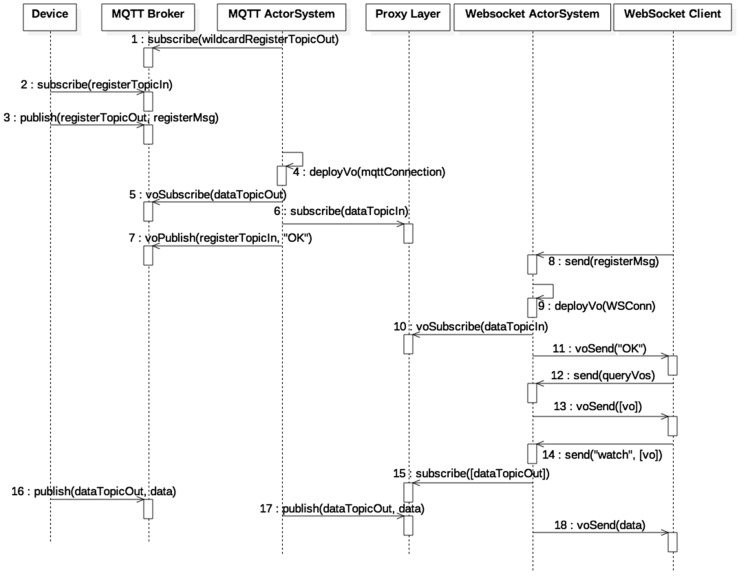
Entity interaction diagram.

In order to transmit data to end nodes of this architecture (Device/SmartGateway and WebSocket Client in Figure 6), we have developed a communication protocol at the application layer that exploits the real-time capabilities of the proposed architecture. The protocol is depicted in Figure 7 and explains the actions conducted by each entity involved in the communication process. Involved entities are:

- Device: A device or smart gateway. In a Smart Grid setting, a smart meter or any other intelligent electronic device can be the Device.
- MQTT Broker: An MQTT server or broker that handles subscriptions and publications and deliveries messages from publishers to subscribers.
- MQTT ActorSystem: An Actor System responsible for translating the MQTT protocol to a homogeneous protocol. It is also responsible for allocating virtual objects by deploying the corresponding actors.
- Proxy Layer: A publish/subscribe system and a database.
- WebSocket ActorSystem: An Actor System responsible for translating the WebSocket protocol to a homogeneous protocol. It is also responsible for allocating virtual objects by deploying the corresponding actors.
- WebSocket Client: For example, a browser that supports the WebSocket protocol.

Entities perform actions by sending asynchronous messages. Actions between entities are represented in Figure 7 by directed arrows and the associated label gives information about the action performed and the involved and exchanged information in those actions. The information shown in parentheses can be seen as variables whose name gives semantic hints about their content. The schematic is explained as follows:

1. In step 1, an actor named MQTT Master that has been initially allocated in the MQTT ActorSystem subscribes to a wildcard topic such as *+/config/out* to receive incoming information about new devices. The plus sign in the wildcard topic means that the subscription is to all topics that match a *string* prepended by */config/out*.
2. A Device which communicates via MQTT initiates a registration process with the MQTT ActorSystem via the MQTT broker. As shown in steps 2 to 7, the Device subscribes to a topic dedicated to configuration purposes, which includes the registration process, and sends a registration request to the MQTT Master. If the registration process is successful, which is the flow shown, the MQTT Master deploys a dedicated actor responsible for handling the messages sent over the configuration topic and the data topic (*dataTopicOut*). The actor also subscribes to the Proxy Layer to receive messages directed to the device (step 6). Finally, the actor responds with an ACK (“OK” message) to the Device communicating that the registration process was successful. The virtual object that represents the Device and associated information is registered in the system’s database (action not shown in Figure 7). Note that the actions prepended with the prefix “vo” (virtual object), (e.g., voSubscribe) correspond to actions performed by a dedicated actor, thus the voSubscribe action is performed by the actor deployed by MQTT Master in step 4. Also, the topic or message used in a “vo” action contains the ID of the virtual object and therefore messages and topics do not collide between Virtual Objects.
3. The WebSocket Client sends a message to the WebSocket ActorSystem, concretely to an actor named WebSocket Master, to initiate a registration process that starts at step 8 and ends at step 10. If successful, the WebSocket Master deploys a new actor which will be responsible for handling the messages sent over the previously established WebSocket connection (WSConn). The subscription process ends at step 11 when the dedicated actor sends and ACK (“OK” message) meaning that the registration process was successful. As with the MQTT registration process, the associated information is saved into the database.
4. Steps 12 and 13, exemplify a WebSocket Client query requesting for available virtual objects the dedicated actor response with appropriate data. The process of the actor querying the database is not shown in Figure 7. Then, in step 14, the WebSocket Client sends a message indicating what virtual objects to watch or subscribe to. Once the dedicated actor receives this information, it subscribes to the corresponding virtual object topics to receive updates of them in step 15. Variables in square brackets “[]” indicate a list of those variables.
5. Finally, steps 16 to 18 show how messages produced by the Device are consumed by the WebSocket Client.

Note that the process explained can be reversed. Once the devices (Device and WebSocket Client) connect to the architecture, both can perform the same actions. In fact, at the software level, the functionalities of both virtual objects are encoded equally (classes in Object Oriented Programming).

### 4.3. Emulation Setting

To implement the model represented in Figure 6:

- Mosquitto has been used as the MQTT server [54].
- Akka [52] has been used through the Scala API as an implementation of the Actor Model in Regions A and B. Note that the Erlang Virtual Machine allows actors to be hot deployed and that the characteristics of the Java Virtual Machine make this functionality harder to implement. Nevertheless, the OSGi framework provides the necessary means to accomplish this task, which could be useful for implementing Software Defined Utilities in the same way as used in Software Defined Networks [1,16]. Both in Erlang and Akka, an actor does not correspond to a system thread, but instead, a few threads, usually as many as the number of cores, are scheduled to process each actor mailbox when new messages arrive in order to optimize resources.
- For the Proxy Layer with the publish/subscribe function, a publish/subscribe cluster implementation has been used with Akka-Cluster.
- The Play Framework [55] has been used as WebSockets and HTTP server.
- MongoDB [56] has been used as the database to store available virtual objects.

In order to emulate the maximum number of network hops and distributed resources to approximate a real scenario, we have used the maximum number of lab resources that are at our disposal. We have used a MacBook Pro to simulate a large number of Devices and two Virtual Machines (VM). Each Virtual Machine is allocated in a different physical computer. Table 2 shows the specifications for each lab resource and the entities/services (Figure 7) allocated in them.

## 5. Experimentation and Results

In this section, we present an analysis of the successfully implemented prototypes that are governed by the techniques analyzed above. Our goal is to analyze the real-time performance of our system and to study how it behaves under different types of load. Concretely, the experiments measure the time interval from the moment the sensor data is sent by the Device or Smart Gateway until it arrives at the WebSocket Client.

The minimum message size includes the message id, the virtual object id, the payload, the unit of measure and the time the value was sensed. With payload or value equal to 0 bytes, the minimum message size is 132 bytes. The message id and virtual object id are raw UUIDs (Universally Unique Identifier) (36 bytes per UUID with hyphens) but an extra compression could be applied by encoding them to Base64 (22 Bytes) or Base85 (20 bytes), although the latter encoding is not URL-safe. The timestamp is represented as a 13-byte string. Finally, the unit of measure is represented as 1 byte and the remaining bytes account for JSON-encoding (JavaScript Object Notation) characters. Extra compression can be achieved using binary formats [57] but it was not within the scope of the project.

The legend in each experiment shows the mean, minimum, and maximum difference from the sending of a message from the Device to the reception of the same message by the WebSocket Client. From now on, we will refer to this difference as ∂t (*timedelta*). Therefore, we define ∂t=rt−st for a message, where rt is the reception time and st is the sending time. We also define:

$$mean=mean\left(\partial t_{i}\right)$$

$$max=max\left(\partial t_{i}\right)$$

$$min=min\left(\partial t_{i}\right).$$

### 5.1. Experiment 1

**Figure 8:**
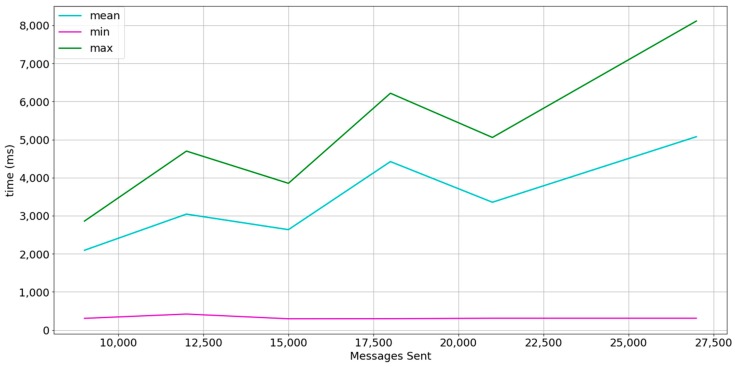
Results in experiment description setting 1 at a 1-s interval between bursts. The line in cyan represents the mean timedelta, the line in magenta represents the minimum timedelta, and the line in green represents the maximum timedelta.

The Dataport-PecanStreet project [58] provides a huge dataset of smart meter data. We have extracted the data from January 2015. The dataset contains data from 633 households and records of energy consumption and production at 15 min interval. Each record contains information about a maximum of 66 smart appliances per household but each household has no more than 15 smart appliances generating smart meter readings. A total of 124,331,328 individual smart meter records are available and 28,257,120 contain energy usage readings. For this reason, the payload size has been fixed to 4 bytes to fit a smart meter reading in each message and load tests are performed until total transmitted messages per burst resemble a scenario in which each household owns 15 smart appliances. As the experiments will be performed in an emulation setting and the goal is to perform stress tests, the interval of 15 min per smart meter reading has not been taken into account. We also perform 3 bursts where each device sends a message. The interval between bursts is of 1 s. Bursts are performed in order to analyze how actor mailboxes behave. Table 3 summarizes the description of the experiment. Figure 8 shows the results.

As shown in Figure 8, the mailboxes of the actors tend to saturate at each sample, but the timedelta of the first message is less than 500 ms. A high percentage of messages are affected by the queue size, as the mean shape is closer to the maximum.

**Figure 9:**
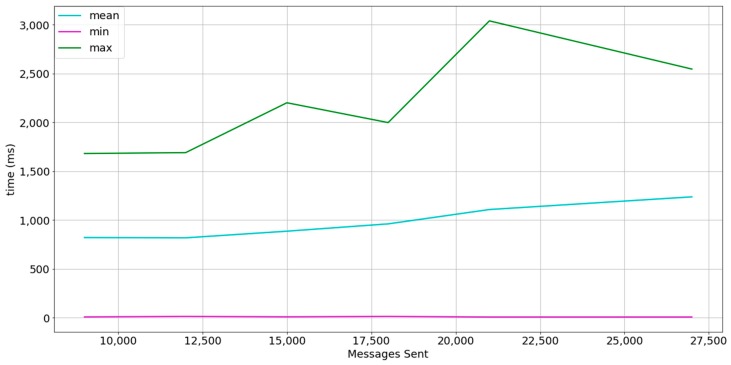
Results in experiment description setting 1 at a 5-s interval between bursts. The line in cyan represents the mean timedelta, the line in magenta represents the minimum timedelta, and the green line represents the maximum timedelta.

Increasing the burst interval to 5 s, smooths the timedelta mean as shown in Figure 9. This means that a higher percentage of timedeltas compared with Figure 8 is closer to the minimum. It also shows that the maximum is 1.3 s approximately, compared to 5 s in Figure 8. The results obtained conform to time needs in non-critical Smart Grid applications [4,16,59].

### 5.2. Experiment 2

The experiment description setting 2 is similar to the experiment description setting 1 but instead of increasing the number of simulated devices, it emulates the aggregation of messages in the payload field. The experiment shows 1000 devices sending messages with payloads that aggregate messages, emulating that each device is a Smart Gateway that aggregates messages. Table 4 summarizes the experiment description. 

**Figure 10:**
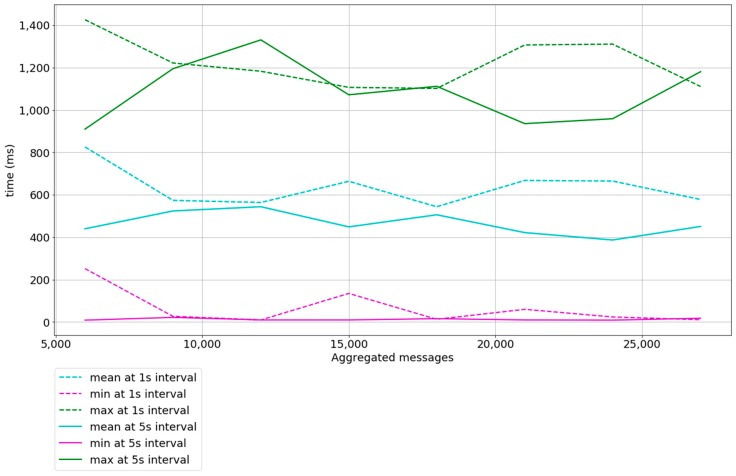
Results in experiment description setting 2 at 1 and 5-s interval between bursts. Dashed lines represent experiments at a 1-s interval between bursts and continuous lines represent experiments at a 5-s interval between bursts. Cyan lines represent the mean timedelta, magenta lines represent the minimum timedelta, and lines in green represent the maximum timedelta.

The results of the experiment are shown in Figure 10. Note that the *x*-axis shows aggregated messages instead of sent messages. For example, the message size of a message that aggregates messages is calculated as:

$$\begin{array}{l} \mathrm{messageSize}\left(\mathrm{aggregatedMessages}\right)\\ \;=\mathrm{baseSize}+\mathrm{aggregatedMessages}*\left(\mathrm{baseSize}+\mathrm{payload}\right) \end{array}$$

where payload=4 bytes, baseSize=132 bytes. If a Smart Gateway sends two aggregated messages, the total payload is calculated as:

messageSize(2)=132+2∗(132+4)=132+272=404 bytes.

The difference of the mean, minimum, and maximum parameters between samples at 1 s interval and 5 s interval are not abrupt, which means that reducing the interval between bursts from 5 s to 1 s does not have a great impact on performance, even with messages that have a payload equal to 1224 bytes. We conclude that our system is capable of delivering 1000 messages aggregating nine messages in their payload within a ∂t<1.42 s at an interval of 1 s between 1000 messages, which is equal to 9000 aggregated messages delivered in less than 1.5 s and at a mean rate of 600 ms. The experiments start when 2 messages are aggregated, since the aggregation of 1 message is nonsense.

### 5.3. Discussion between Experiments 1 and 2

**Figure 11:**
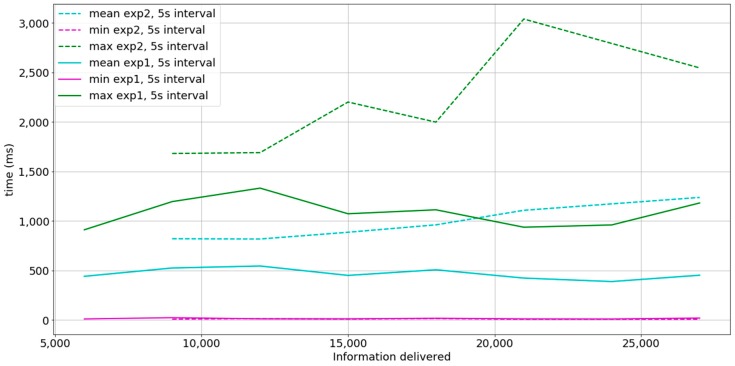
Comparison between experiment 1 and experiment 2 at a 5-s interval between bursts. *x*-axis equals to the total information delivered and refers to both individual messages at experiment 1 and aggregated messages at experiment 2. Dashed lines represent measurements in experiment 2 at a 5-s interval between bursts. Continuous lines represent measurements in experiment 1 at a 5-s interval. Cyan lines indicate the mean timedelta, magenta lines indicate the minimum timedelta, and green lines represent the maximum timedelta.

In Figure 11 a comparison between experiments 1 and 2 with the description setting where the time between bursts is 5 s is shown. The mean timedelta obtained in both experiments shows that a better performance can be achieved by aggregating messages (experiment 1). The slope of the mean function in experiment 1 is approximately 0 and the slope of the mean function in experiment 2 is positive. No messages were lost in either experiment 1 nor 2. While results in experiment 1 conform to time needs of non-critical Smart Grid applications, results in experiment 2 conform to some of time needs in critical Smart Grid applications [4,59,60]. We also conclude that message aggregation is preferred when possible, as it reduces the overheads of the entire system.

### 5.4. Web of Energy, Proof of Concept

**Figure 12:**
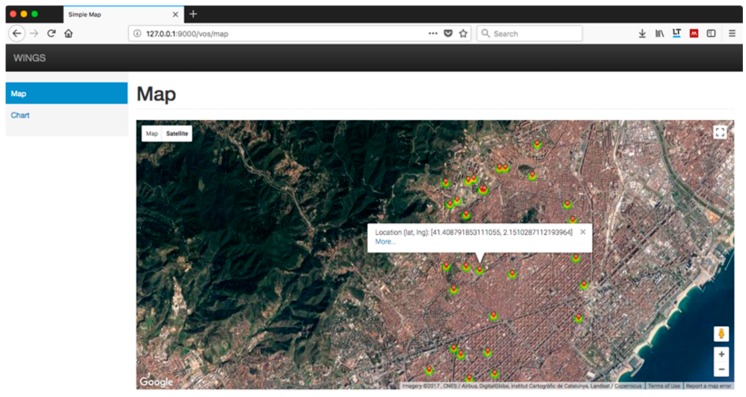
Graphic representation of sensor location.

To conclude the experiments, we directed our efforts to developing a web portal to visualize the data available through the HTTP and the WebSocket protocol, which prototypes the WoE. 1000 devices have been simulated, emitting synthetic smart meter data every 5 s. These sensors are distributed throughout the city of Barcelona. Figure 12 shows one of the functionalities of the system, a heat map updated every 5 s in near real-time. However, Figure 12 only shows a few of those sensors because the web browser performance is rapidly decreased as more and more objects need to be painted. A possible solution could be to aggregate smart meter readings and show the aggregation value in the heat map.

**Figure 13:**
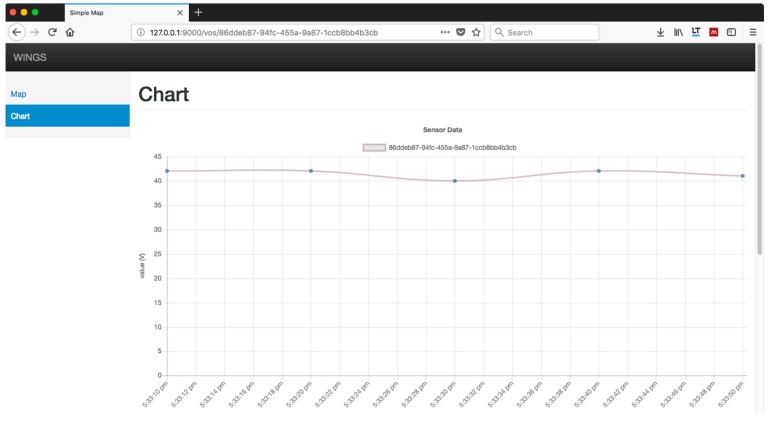
Chart showing the synthetic values emitted by a sensor every 5 s.

Figure 13 shows the result of consulting the value of one of these sensors by clicking in the “More…” link shown in Figure 12. The chart label corresponds to the universal unique identifier (UUID) of the sensor. The values in the vertical axis correspond to the measurements obtained by the sensor. This simulated sensor reads voltage, so a “V” inside the parentheses is displayed. The values in the horizontal axis show the time at which the voltage reading is obtained, every 5 s. The data shown updates in near real-time.

The application is designed with the ability to interact with each of these sensors, although in this initial version only the query of values is allowed. All queries are executed in near real-time and without delays, despite the large volume of data. This confirms that the design of the prototype is consistent and allows the extension of the rest of the WoE.

### 5.5. Discussion and Conclusions

A WoE prototype based on the Actor Model has been implemented. Our goals were: (i) to address some of the key issues related to ICTs in Smart Grid systems while demonstrating the benefits of the Actor Model in such ICT—Smart Grid use cases and (ii) to implement a prototype for further research. The Smart Grid can be seen as a specific Internet of Things application, with its own constraints and requirements. Specifically, we have addressed the heterogeneity of protocols by means of the WoT and some of the near real-time requirements of Smart Grid ICT systems. We have created Virtual Objects, a common abstraction in IoT scenarios, by means of the Actor Model. Each Virtual Object is represented by at least one actor, a lightweight reactive agent. Heterogeneity is addressed in the prototype system by the capacity of each implemented actor to translate heterogeneous protocols (MQTT, for example) to web protocols (HTTP or WebSocket). Performance tests show that the initial model meets some of the time needs of time-critical Smart Grid ICT applications when Virtual Objects aggregate several smart meters (messages that aggregate messages). In such cases, the timedelta mean slope remains neutral even at bursts of 9000 aggregated messages at a 1-s interval between bursts. Tests involving more devices have to be carried out in order to explore the scalability possibilities of the system. Note that system is flexible in that the actors are allocated/deployed when needed. Experiments also show that the system is suitable for non-time-critical Smart Grid traffic when messages can’t be aggregated before reaching the prototype system. Finally, a simple web-based interface to visualize sensor location and sensor readings in near real-time has been implemented as the WoT interface to the Smart Grid, the Web of Energy.

The results encourage us to keep investigating in this direction. We acknowledge that our system and experiments do not cover all of the scalability and flexibility requirements in Smart Grid ICT systems but we have presented the benefits of the Actor Model for such systems and developed an initial prototype for further research and development.

## 6. Conclusions

While the overestimations on the initial prediction of the volume of internet connections were acknowledged and duly rectified, the fact that our society is becoming increasingly connected to the internet is undeniable. This acceleration has been promoted thanks to the advances of both silicon, which makes it possible to embed increasingly smaller computing units in everyday devices, and advances in low-power wireless protocols. At the same time, internet and web applications targeted at controlling and managing internet-connected devices are being continually improved. We are being encouraged to integrate internet-connected devices into our lives, where social network services serve as the easy-to-use interface between people and devices.

It is obvious that the Web of Things is continually extending to different application domains. Nevertheless, in order to continue this trend, it is essential that the software applications involved are built following guidelines that guarantee their performance, accessibility, and high availability. In this sense, an overall development model agreed upon and approved by different authors is becoming increasingly specific. 

In this paper, the term Web of Energy is used to refer to the critical features that an architecture of the Web of Things must fulfill to be applied to the domain of Smart Grids. We highlight the requirements related to the implementation of smart functions, such as the distribution, resilience and self-healing of the system as well as the difficulties of renewing the traditional energy generation and management systems, both in terms of device and the software, which are generally interrelated. Given those challenges, the use of the Actor Model is proposed to overcome them. This paradigm is designed specifically to perform the modeling of concurrent and distributed systems, thus directly improving the inherent characteristics of distributed systems. In this sense, a proposal of middleware architecture using this paradigm for the creation of a ubiquitous sensor network has been presented in this paper. 

## 7. Future Work

The future work to be done is focused on two different aspects. On the one hand, we want to expand the WoE application, providing it with more functionalities and allowing us to interact with the devices. We also want to integrate different types of sensors, with unequal capacities and performances.

On the other hand, characteristics of scalability, discoverability, and interoperability are only partially tackled by the WoT as it serves as the abstraction layer that comprises technologies that enable homogeneous communication among devices by means of HTTP and semantic web ontologies. However, a global IoT or WoT, the latter being an IoT empowered by web technologies, still has scalability and discoverability issues. As the number of Internet-connected devices grows, the lack of global scalability and discoverability functionalities will become more evident. In this regard, a novel IoT paradigm, the Social Internet of Things (SIoT) is gaining momentum.

In this novel paradigm, communication between social virtual objects becomes mandatory and the amount of messages exchanged between devices is expected to increase. Several works aim to provide example scenarios where SIoT can be leveraged [61,62,63], to name a few. In this sense, we highlight [63], where the authors use thing-to-thing social relationships to encourage communication to optimize energy usage by the Heat, Ventilation, Air Conditioning (HVAC) system in their laboratory. Results show that the comfort-aware HVAC system ensures users’ thermal comfort, while at the same time reducing energy costs with respect to static and traditional methods. An ongoing challenge for us is the study of the implications of reactive, asynchronous communication together with computation distribution properties of the Actor Model in a SIoT system, in terms of performance and how these abstractions facilitate the development of this kind of applications. Further work on this subject encompasses the creation of proper simulation tools or modules to facilitate the simulation of SIoT enabled nodes and networks at multiple network layers (e.g., applications installed on top of constrained devices and fog or cloud applications).

## Figures and Tables

**Table 1:** WoT/IoT protocols.

	HTTP	WebSocket	CoAP	MQTT	XMPP	AMQP	DDS
Topology	Req/Resp	Two-way, realtime	Req/Resp	Pub/Sub	Pub/Sub and Req/Resp	Pub/Sub	Pub/Sub
Architecture	P2P	P2P	P2P	Broker	P2P	P2P or Broker	Global Data Space
Transport Level	TCP	TCP	UDP	TCP	TCP	TCP	TCP/UDP
Encryption	TLS	TLS	DTLS	TLS	TLS	TLS	TLS/DTLS
Authentication	TLS	TLS	DTLS	User/Pass	TLS	SASL	TLS/DTLS
QoS	-	-	Confirmable	3	-	3	23

**Table 2:** Resources used in the emulation setting and service allocation.

Resource	Specifications	Services
VM 1	2 × 2.4 GHz Intel Xeon (4 cores per processor), 4 GB RAM	Mosquitto, MQTT ActorSystem
VM 2	3 × 2.4 GHz Intel Xeon (4 cores per processor), 6 GB RAM	Proxy Layer, HTTP/WebSocket Server and ActorSystem
MacBook Pro	2.7 GHz Intel Core i7 (2 cores per processor), 8 GB RAM	MQTT Devices, WebSocket Client

**Table 3:** Experiment 1 description setting.

Variable	Value
Devices	{1k, 2k, 3k, 4k, 5k, 6k, 7k, 9k}
Payload Size (bytes)	{4}
Burst Interval (ms)	{1000, 5000}
Bursts	{3}

**Table 4:** Experiment 2 description.

Variable	Value
Devices	{1000}
Payload Size (bytes)	{272, 408, 544, 680, 816, 952, 1224}
Burst Interval (ms)	{1000, 5000}
Bursts	{3}
